# A Vignette-Based Educational Intervention to Reduce Neurophobia and Improve Knowledge for the UK Medical Licensing Assessment: A Prospective Pilot Study

**DOI:** 10.7759/cureus.95205

**Published:** 2025-10-23

**Authors:** Zayna S Ahmed, Nigel Mason, Clare Galton

**Affiliations:** 1 Neurology, Ipswich Hospital, East Suffolk and North Essex NHS Foundation Trust, Ipswich, GBR; 2 Quality Improvement, Ipswich Hospital, East Suffolk and North Essex NHS Foundation Trust, Ipswich, GBR

**Keywords:** fourth-year medical student education, medical education curriculum, medical school education, neurophobia, single best answer, the national model core curriculum for undergraduate medical education, undergraduate curriculum

## Abstract

Introduction: Neurophobia, an aversion to clinical neurology, undermines medical education and may affect patient safety. The UK Medical Licensing Assessment (MLA) emphasizes vignette-based Single Best Answer (SBA) items, requiring practice in applied clinical reasoning. We developed a compact, MLA-aligned educational intervention to reduce neurophobia by improving confidence and knowledge.

Methods: Using the Model for Improvement, we produced a 34-page booklet and delivered a 90-min, resident-led, interactive session across two plan-do-study-act cycles at a district general hospital (QIP: 25-586). Sixteen students participated (two cohorts, n=8 each), completing paired pre- and post-session assessments. While limited by a small sample size, this pilot supports proof of concept and warrants multicenter evaluation to strengthen generalizability. The primary outcome was self-rated confidence (five-point Likert). The secondary outcome, assessed in cycle 2, was knowledge measured by a six-item SBA test. Wilcoxon signed-rank tests compared paired scores; effect sizes were reported as $\left| r \right| = \frac{\left| Z \right|}{\sqrt{N}}$. Cronbach’s alpha assessed reliability.

Results: Both cycles produced significant confidence gains. In cycle 1, median confidence rose from 2.5 (IQR: 2.0-3.0) to 4.0 (IQR: 4.0-4.25) (p<0.05; |r|=0.90). In cycle 2, confidence rose from 3.0 (IQR: 2.0-3.0) to 5.0 (IQR: 4.75-5.0) (p<0.01; |r|=0.89). In cycle 2, mean SBA scores improved from 25.0% (SD: 8.33) to 97.9% (SD: 5.5) (p<0.01; |r|=0.90). The SBA tool showed acceptable reliability (Cronbach’s α=0.76). Five of six items reached 100% correct post-session; the sixth reached 87.5%.

Conclusion: A single, low-resource, MLA-aligned session significantly improved medical students’ confidence and objective knowledge in neurology. Conducted as a prospective pilot study using QI methodology, it demonstrates proof of concept and supports wider evaluation including multicenter trials and delayed retention testing to mitigate neurophobia and strengthen workforce readiness.

## Introduction

Clinical neurology is widely recognized as one of the more demanding areas of medicine. Students often describe how its reliance on precise anatomical localization, the complexity of neurophysiological concepts, and the habit of "lesion thinking" combine to form a cognitive barrier that erodes both confidence and competence [1,2]. The term neurophobia captures this cluster of anxieties and has been linked to avoidance behaviors and suboptimal learning outcomes across several countries [3-5].

At the same time, high-stakes assessments, such as the UK Medical Licensing Assessment (MLA), increasingly place an emphasis on vignette-based clinical reasoning and Single Best Answer (SBA) formats. These assessments reward the integration and application of knowledge rather than rote recall. However, the teaching students receive during neurology placements, particularly in district general hospitals, often remains fragmented and opportunistic, leaving them without the structured frameworks needed for success in both exams and early clinical practice [6,7].

Resident doctors play a significant role in bridging this gap. They are often best placed to translate complex neurological ideas into practical heuristics, though their capacity to provide structured teaching is frequently limited by clinical pressures [8]. Recognizing these systemic and pedagogical shortcomings, we registered a quality improvement project (QIP: 25-586) with our institutional QI office. Our Specific, Measurable, Achievable, Relevant, Time-bound (SMART) aim was precise, i.e., to increase the average self-assessed confidence score of medical students in neurology from 2.5/5.0 to 4.0/5.0 by May 1, 2025. This aim was implemented within the Model for Improvement framework, employing iterative plan-do-study-act (PDSA) cycles to refine both delivery and measurement.

The primary objective was to improve medical students’ self-rated confidence in neurology through an MLA-aligned, vignette-based session, and the secondary objective assessed in the second PDSA cycle was to evaluate objective knowledge gain using a six-item SBA test. By turning clinical reasoning into brief, case-based practice, the intervention sought to reduce neurophobia and better prepare students for both licensure assessments and day-to-day clinical work.

## Materials and methods

This prospective quality improvement project was conducted between January and April 2025 at a district general hospital in the East of England. The study employed the Model for Improvement and two sequential plan-do-study-act (PDSA) cycles to iteratively develop and refine the intervention [8,9]. The project was registered with the East Suffolk and North Essex NHS Foundation Trust (ESNEFT) Quality Improvement Office (QIP: 25-586). As a service evaluation aimed at improving local educational delivery, the project was exempt from formal review by the UK NHS Health Research Authority’s Research Ethics Committee. All participants provided written informed consent for the anonymized use of their data for evaluation and publication. Data handling complied fully with General Data Protection Regulation (GDPR) standards.

Sixteen medical students on core clinical neurology placements were recruited across two consecutive eight-week blocks. The sample size was determined by placement cohort availability (opportunistic convenience sample). Each PDSA cycle included eight students. Participation was voluntary, and all invited students elected to take part, yielding a 100% paired response rate for all assessments.

The intervention was a 90-min, interactive, resident-facilitated teaching session supported by a purpose-designed 34-page booklet, Neurology for Medical Students. The booklet summarized high-yield concepts and included MLA-style vignettes with Single Best Answer (SBA) questions and fully explained answer rationales. Content selection prioritized syndromes and decision points relevant to routine clinical practice and MLA blueprint topics. Cycle 1 consisted of the initial booklet and facilitated session. Cycle 2 incorporated refinements based on participant feedback, including a portable clinical aid summarizing key neurological examination findings and a higher proportion of test-style questions to mirror examination conditions [10]. The full session outline and contents of the 34-page booklet are provided in appendix 1, and the pre- and post-session confidence and knowledge survey tools are provided in appendix 2.

The primary outcome was self-assessed confidence in neurology, recorded using a five-point Likert scale administered immediately before and after each session. The secondary outcome was objective knowledge, measured in both cycles using a six-item SBA test administered immediately pre- and post-session. The SBA items, authored by the lead investigator and reviewed by a consultant neurologist for content validity, were thematically aligned with the session but distinct from the worked examples in the booklet. An example item is provided to illustrate the question format and level of reasoning assessed. A 62-year-old woman presents with stiffness and weakness when climbing stairs. Examination reveals mild upper-arm weakness, hip flexion 4/5 bilaterally, quadriceps fasciculations, brisk ankle reflexes, and a positive Babinski sign. Sensory examination is normal. What is the most likely diagnosis? (A) Multiple sclerosis, (B) myositis, (C) diabetic neuropathy, (D) motor neuron disease, or (E) myasthenia gravis.

As the data were paired and not assumed to follow a normal distribution, Wilcoxon signed-rank tests were used to compare pre- and post-intervention scores. Effect sizes were reported as absolute values ($|r| = \frac{|Z|}{\sqrt{N}}$) for interpretability. Statistical significance was defined as p<0.05. The internal consistency of the six-item SBA test was evaluated using Cronbach’s alpha, with α≥0.70 considered acceptable [11]. Analyses were performed using IBM SPSS Statistics version 28 (Chicago, IL: IBM Corp.).

## Results

All 16 participants completed paired pre- and post-session measures. The intervention produced substantial and statistically significant improvements in confidence and objective knowledge, as detailed in Table 1. Item-level question performance and teaching content converted into question and answer (Q&A) format are presented in appendix 3 (table in appendix 3).

**Table 1:** Summary of pre- and post-intervention outcomes for confidence and knowledge (all scores out of 5).

Cohort (n=8)	Outcome measures	Pre-intervention (median/mean)	Post-intervention (median/mean)	Test statistic (Z)	p-Value	Effect size (r)
Cycle 1	Confidence, median (IQR/5)	2.5 (2.0-3.0)	4.0 (4.0-4.25)	-2.53	<0.05	1.00
Cycle 2	Confidence, median (IQR/5)	3.0 (2.0-3.0)	5.0 (4.75-5.0)	-2.52	<0.01	0.89
Cycle 2	Knowledge, mean%, (SD) → (normalized to a 5-point scale)	25.0% (SD 8.3) ≈ 1.25/5	97.9% (SD 5.5) ≈ 4.90/5	-2.55	<0.01	-

**Figure 1:**
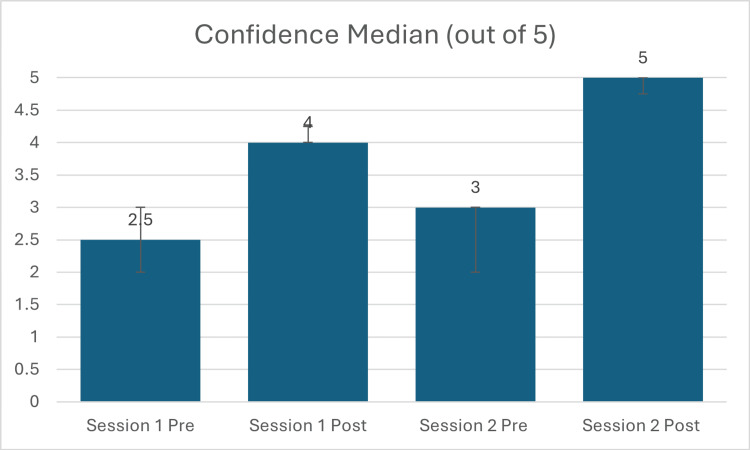
Change in medical student self-assessed confidence in neurology following the educational intervention. Median confidence scores (out of 5, with IQR error bars) for cycle 1 (n=8) and cycle 2 (n=8), before and after the session. Both increases were statistically significant.

In cycle 1 (n=8), the median self-assessed confidence score (out of 5) increased from 2.5 (IQR: 2.0-3.0) to 4.0 (IQR: 4.0-4.25). This change was statistically significant (p<0.05) and associated with a large effect size (|r|=1.00). In cycle 2 (n=8), median confidence (out of 5) rose from 3.0 (IQR: 2.0-3.0) to 5.0 (IQR: 4.75-5.0) (p<0.01; |r|=0.89). These results are illustrated in Figure 1.

**Figure 2:**
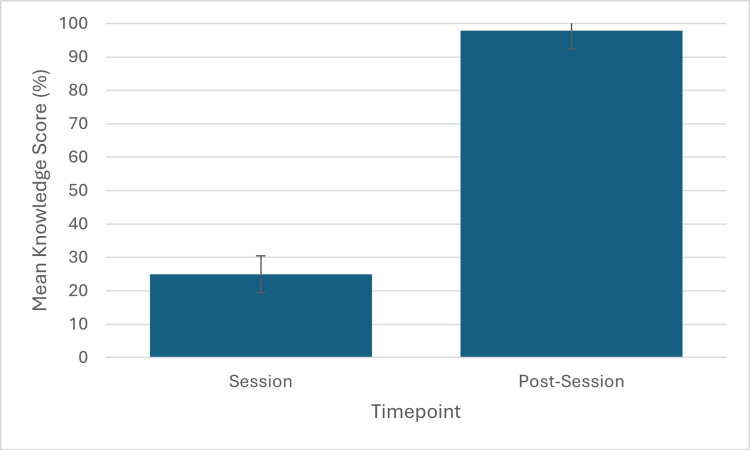
Improvement in objective knowledge scores in cycle 2. Mean percentage scores (with SD error bars) on a six-item SBA test: pre-session 25.0% (SD: 8.33) vs. post-session 97.9% (SD: 5.5), p<0.01. SBA: Single Best Answer

Objective knowledge, measured in cycle 2 (n=8), increased from a mean SBA score of 25.0% (SD: 8.33) pre-session to 97.9% (SD: 5.5) post-session. This represented a highly significant improvement (p<0.01) with a large effect size (|r|=0.90). The six-item SBA instrument illustrated acceptable internal consistency (Cronbach's α=0.76). An item-by-item analysis demonstrated pre-session correct response rates ranging from 12.5% to 37.5%. Post-session, performance on five of the six items reached 100% correct, while the sixth item was answered correctly by 87.5% of participants (Table 2). These findings are illustrated in Figure 2.

**Table 2:** Item analysis of SBA questions pre- and post-intervention (cycle 2, n=8). SBA: Single Best Answer

SBA question item	Pre-intervention (% correct)	Post-intervention (% correct)
SBA Q1	37.5	100.0
SBA Q2	25.0	100.0
SBA Q3	12.5	100.0
SBA Q4	37.5	100.0
SBA Q5	12.5	87.5
SBA Q6	25.0	100.0

Qualitative feedback from free-text surveys consistently emphasized the session's interactivity and strong alignment with examination practice. All the students reported that the SBA format encouraged active engagement and helped them understand the reasoning behind correct and incorrect answers, while the accompanying booklet served as a portable reference that reinforced key concepts. As outcomes were measured immediately post-session, the reported improvements represent short-term learning gains and should be interpreted as indicative of immediate efficacy rather than long-term retention.

## Discussion

Brief, MLA-aligned vignette practice produced significant, immediate gains in both confidence and objective knowledge among medical students. Given the pilot design and immediate post-testing, these data should be interpreted as proof of concept that warrants controlled, multisite evaluation.

Neurophobia persists because neurology uniquely obliges learners to synthesize complex anatomy, physiology, and clinical signs into localization-based reasoning [1,2]. In district general hospitals, teaching is often opportunistic rather than organized, and resident doctors face competing clinical demands that limit consistent instruction [7,8]. Our intervention operationalized lesion-based and syndromic reasoning utilizing MLA-style vignettes that facilitated stepwise localization and differential prioritization. This use of context-rich problems aligns with established principles for the promotion of clinical reasoning skills in trainees [12]. This alignment likely explains the rapid translation into objective test performance, as learners practiced the exact cognitive tasks that the assessments evaluate.

Addressing neurophobia is a matter of patient safety and workforce preparedness. For a resident doctor, failing to recognize a hemiparetic stroke at the door of the emergency department is not a theoretical risk but a daily hazard. Similarly, hesitation in identifying a first seizure or an acutely raised intracranial pressure can carry profound consequences for patient outcomes. Gaps in neurology confidence, therefore, contribute not only to delayed recognition of emergencies but also to avoidance of neurology as a career choice at a time when the specialty faces workforce shortages [3-5]. By aligning teaching activities with assessment requirements - a principle known as constructive alignment - this intervention practises the reasoning skills required by both the MLA and safe clinical practice [13,14]. From a cognitive load theory perspective, the booklet reduced extraneous cognitive demands by consolidating concepts, while the SBAs provided structured, deliberate practice [15]. These educational principles likely contributed to the large effect sizes we observed. The small-group format enabled personalized feedback and open discussion, which may have amplified the effect [16].

This pragmatic model pairs near-peer facilitation with standardized, portable materials that are inexpensive to reproduce and can be scaled by running parallel small groups or embedding the booklet in curricula. The intervention's advantages over resource-intensive alternatives are notable, as high-fidelity simulation requires infrastructure and staffing commitments that many institutions will have difficulty sustaining [17].

This study’s limitations must be acknowledged. The sample size was small and from a single center, limiting generalizability. Knowledge was assessed immediately post-intervention, by design, to capture the direct educational impact of a single exposure; however, this approach does not evaluate long-term retention - a critical determinant of durable learning, as single-session gains often decay without reinforcement or retrieval practice [18,19]. The absence of a control group means that a testing effect cannot be ruled out, although the magnitude of improvement suggests this is an incomplete explanation [20]. In addition, volunteer bias may have influenced the results, as students with a pre-existing interest in neurology might have been more engaged. The possibility of partial overlap between teaching and testing content, as well as ceiling effects on the confidence scale, could also have inflated post-session scores. These limitations are common in early QI work, and this project therefore serves as a robust pilot demonstrating feasibility and effect magnitude [21-23].

The following steps include a cluster-randomized, multisite trial and delayed retention testing at 4-12 weeks to evaluate durability. Embedding the intervention into the formal curriculum would enable longitudinal evaluation of both educational outcomes and, eventually, impact on patient care, such as earlier recognition of neurological emergencies by resident doctors [24,25].

## Conclusions

Neurophobia undermines medical education and patient safety, leaving students underconfident and ill-prepared for clinical practice. This pilot study demonstrates that a single, resident-led session, supported by a vignette-based booklet aligned to MLA SBA formats, can deliver large and statistically significant gains in both confidence and objective knowledge. Developed and refined using QI methodology, the intervention is compact, low-cost, and reproducible. Its alignment with national assessment standards positions it as a practical model for broader use. Future work should extend to multicenter evaluations and delayed retention testing to confirm its long-term impact and support formal curricular integration.

## Appendices

Appendix 1

**Table 3:** Confidence and feedback survey (tabular).

Item no.	Survey question	Response format
1	How helpful did you find the teaching session?	5-point Likert: 1 (not very helpful) - 5 (very helpful)
2	How confident did you feel about the topic before the teaching session?	5-point Likert: 1 (not very confident) - 5 (very confident)
3	How confident did you feel about the topic after the teaching session?	5-point Likert: 1 (not very confident) - 5 (very confident)
4	What did you like about the teaching session?	Free-text (open)
5	What are the areas we could improve on?	Free-text (open)
6	How high-quality do you think the session was?	5-point Likert: 1 (poor quality) - 5 (excellent quality)

Appendix 2

**Table 4:** Pre- and post-session knowledge survey (SBA instrument). SBA: Single Best Answer

Item no.	Vignette stem (abridged)	Options (A-E)
1	62-year-old woman; stiffness and difficulty climbing stairs; mild proximal weakness, quadriceps fasciculations; brisk reflexes; Babinski; normal sensation. Most likely diagnosis?	(A) Multiple sclerosis, (B) myositis, (C) diabetic neuropathy, (D) motor neuron disease, (E) myasthenia gravis
2	70-year-old man with AF; dizziness then pallor and brief jerks; rapid recovery. Most likely diagnosis?	(A) Vasovagal syncope, (B) orthostatic hypotension, (C) cardiogenic syncope, (D) transient ischemic attack, (E) seizure
3	30-year-old woman; recurrent optic neuritis; current severe attack. Best acute treatment?	(A) Oral prednisolone, (B) IV methylprednisolone, (C) plasmapheresis, (D) beta-interferon, (E) azathioprine
4	60-year-old man with alcohol misuse; active generalized seizure in ED. Most appropriate initial action?	(A) IV access + thiamine + 50% glucose, (B) start clock and observe, (C) PR lorazepam, (D) IV access + IV lorazepam, (E) call 2222 (anesthetics)
5	28-year-old woman; left arm tingling + visual blurring; similar episode 7 months ago. Most appropriate investigation?	(A) CT head with contrast, (B) CT head without contrast, (C) MRI brain and spine with contrast, (D) MRI brain without contrast, (E) MRI whole spine without contrast
6	Young overweight woman; headache; normal CT; LP opening pressure 30 cm H₂O; papilledema. Most appropriate treatment?	(A) Acetazolamide, (B) aspirin, (C) clopidogrel, (D) gabapentin, (E) dexamethasone

Appendix 3

Summary of Teaching Material (Booklet and PowerPoint Converted to Q&A Table Format)

The teaching content was originally developed and delivered using the following two formats: (1) a booklet titled High-Yield MLA Neurology for Medical Students and (2) an accompanying PowerPoint presentation covering the same material. For appendix purposes, the educational content has been reformatted into a question-and-answer (Q&A) table. The clinical vignettes, answers, and explanations remain unchanged. Only the layout has been adapted to improve clarity and facilitate review.

**Table 5:** Summary of high yield neurology teaching material (booklet+PowerPoint content converted into Q&A format). UMN: upper motor neuron; LMN: lower motor neuron; PMA: primary muscular atrophy; FTD: fronto-temporal dementia; ALS: amyotrophic lateral sclerosis; EMG: electromyography; NCS: nerve conduction studies; NIV: non-invasive ventilation; PEG: percutaneous endoscopic gastrostomy; TIA: transient ischemic attack; UL/LL: upper limb/lower limb; CP/MS: cerebral palsy/multiple sclerosis; IVIG: intravenous immunoglobulin; NICE: National Institute for Health and Care Excellence; STAT: Statim (immediately); BP: blood pressure; CNS: central nervous system; NMO: neuromyelitis optica; AQP4 Abs: anti-aquaporin-4 antibodies; GBS: Guillain-Barré syndrome; CSF: cerebrospinal fluid; Q&A: question and answer PowerPoint (Redmond, WA: Microsoft Corp.)

Questions	Options (A-E)	Answer	Explanation
62-year-old woman with stiffness, weakness climbing stairs, fasciculations, brisk reflexes, positive Babinski, and normal sensation	(A) Multiple sclerosis, (B) myositis, (C) diabetic neuropathy, (D) motor neuron disease, (E) myasthenia gravis	(D) Motor neuron disease	Presents with both UMN and LMN signs. Associated with FTD. Types: ALS, progressive bulbar palsy, PLS (UMN only), PMA (LMN only). Diagnosis: EMG, NCS. Management: riluzole, NIV, baclofen, PEG feeding
75-year-old man with AF, brief syncope after dizziness and “lights dimming.” Wife witnessed jerks	(A) Vasovagal syncope, (B) orthostatic hypotension, (C) cardiogenic syncope, (D) TIA, and (E) seizure	(B) Orthostatic hypotension	Due to reduced cerebral perfusion on standing. Differentiate from vasovagal (emotional/straining triggers), cardiogenic syncope (arrhythmia, aortic stenosis), seizures (aura, incontinence, tongue biting), and TIA (rare cause)
Patient with asymmetrical weakness, circumduction gait, and flexed arm posture	(A) Hemiplegic gait, (B) scissoring gait, (C) high-stepping gait, (D) spastic gait, and (E) stomping gait	(A) Hemiplegic gait	UMN lesion (stroke, tumor, abscess). Flexors are stronger in UL; extensors are stronger in LL. Circumduction due to spasticity. Differs from scissoring gait (bilateral spasticity, CP/MS)
23-year-old Asian woman, bilateral optic neuritis, transverse myelitis, and facial pain	(A) Oral prednisolone, (B) IV methylprednisolone, (C) plasmapheresis, (D) beta-interferon, (E) azathioprine	(C) Plasmapheresis	Neuromyelitis optica (Devic’s disease). Similar to MS but with bilateral optic neuritis and longitudinally extensive cord lesions. Acute: IV methylpred + plasmapheresis. Long-term: azathioprine, rituximab, IVIG, cyclophosphamide
74-year-old woman, 2-hour episode of expressive dysphasia, now resolved	(A) Aspirin 300 mg STAT+specialist review within one week, (B) specialist review within two weeks, (C) initiate statin+BP therapy as outpatient, (D) aspirin 300 mg STAT+specialist review within 24 hours, and (E) specialist review within 24 hours only	(D) Aspirin 300 mg STAT+specialist review within 24 hours	Suspected TIA. Immediate aspirin and urgent review within 24 h (NICE). Other options (review in one week/two weeks, statins, BP control) are insufficient acutely
36-year-old alcoholic man seizing in A&E	(A) Gain IV access+thiamine+50% glucose, (B) Start the clock, (C) Give PR diazepam, (D) Gain IV access+IV lorazepam E. Call 2222 for anesthetic assistance	Sequential management approach: (1) start clock → (2) PR diazepam (if no IV access) → (3) IV lorazepam (if access) → (4) thiamine+glucose → (5) escalate to anesthetics if refractory	Priorities: time the seizure, give benzodiazepines, correct hypoglycemia, and prevent Wernicke’s (thiamine). Escalate if >5 min/refractory (status epilepticus)
24-year-old woman, clumsy, stumbling, tingling legs	(A) Stool sample, (B) MRI head, (C) electromyography, (D) nerve conduction studies, and (E) CSF serum electrophoresis	(B) MRI head	Best test for suspected MS (periventricular demyelination). Differentials: GBS (NCS/EMG, stool sample for Campylobacter), NMO (AQP4 Abs), CNS inflammation (oligoclonal bands)
31-year-old woman, previous optic neuritis, now arm numbness/tingling	(A) CT head with contrast, (B) CT head without contrast, (C) MRI brain and spine with contrast, (D) MRI brain without contrast, and (E) MRI whole spine without contrast	(C) MRI brain and spine with contrast	McDonald criteria: ≥2 clinical episodes disseminated in time and space. MRI with contrast confirms acute inflammatory lesions. CT not useful
2-month-old baby, fever, irritability, poor feeding, non-blanching rash, LP: ↑WCC (neutrophils), ↑protein	(A) IV acyclovir, (B) IV cefotaxime+IV amoxicillin, (C) IV cefotaxime, (D) supportive management, and (E) IV amoxicillin	(B) IV cefotaxime+IV amoxicillin	Bacterial meningitis (<3 months). Add amoxicillin for Listeria coverage. Supportive management alone or antivirals are inappropriate
Young overweight woman, headache, CT normal, LP opening pressure 30 cm H₂O, papilledema	(A) Acetazolamide, (B) aspirin, (C) clopidogrel, (D) gabapentin, and (E) dexamethasone	(A) Acetazolamide	Idiopathic intracranial hypertension. Carbonic anhydrase inhibitor - reduces CSF production and intracranial pressure
